# Crystal structure of Ag_3_Dy_2_(NO_3_)_9_ and qu­anti­tative comparison to isotypic compounds

**DOI:** 10.1107/S2056989023004747

**Published:** 2023-06-02

**Authors:** Wilhelm Klein

**Affiliations:** a Technical University of Munich, School of Natural Sciences, Department of Chemistry, Lichtenbergstrasse 4, 85747 Garching, Germany; University of Kentucky, USA

**Keywords:** crystal structure, dysprosium, silver, nitrate

## Abstract

Single crystals of Ag_3_Dy_2_(NO_3_)_9_ (tris­ilver didysprosium nona­nitrate) were obtained from a mixture of AgNO_3_ and Dy(NO_3_)_3_·5 H_2_O. The new compound crystallizes in space group *P*4_1_32 (No. 213) with *a* = 13.2004 (4) Å, *V* = 2300.2 (2) Å^3^, *Z* = 4. The Ag and Dy cations are coordinated by five and six bidentate nitrate anions, respectively. Ag_3_Dy_2_(NO_3_)_9_ is isostructural to several compounds that include alkali metals or ammonium and lanthanide cations, but silver and dysprosium are included for the first time and feature the smallest ion radii observed for this structure type to date. Crystal structures of isotypic compounds are compared.

## Abstract 2

The crystal structure of the title compound and its particular relation to isotypic compounds is considered.

## 1. Chemical context

Double nitrates of alkali metals and lanthanides of the composition *A*_3_ *Ln*_2_(NO_3_)_9_ have been found to crystallize in the chiral space groups *P*4_1_32 and *P*4_3_32 (Wickleder, 2002 ▸). After the first finding of K_3_Pr_2_(NO_3_)_9_ by Carnall *et al.* (1973 ▸), this structure type has been observed in several compounds, to date with K (Carnall *et al.*, 1973 ▸; Vigdorchik *et al.*, 1992 ▸; Guillou *et al.*, 1995 ▸; Gobichon *et al.*, 1999 ▸), Rb (Vigdorchik *et al.*, 1990 ▸; Manek & Meyer, 1993*a* ▸; Guillou *et al.*, 1996 ▸), and NH_4_ (Manek & Meyer, 1992 ▸) as ’*A*′ cations for the lighter lanthanides La–Sm, and also detached examples with Na at the ’*A*′ site (Stockhause & Meyer, 1997 ▸; Luo & Corruccini, 2004 ▸) and Eu (Manek & Meyer, 1992 ▸), Gd (Manek & Meyer, 1992 ▸; Luo & Corruccini, 2004 ▸), and even Bi (Goaz *et al.*, 2012 ▸) at the lanthanide site have been reported. The compounds are typically synthesized by dissolving the lanthanide oxides or nitrates in melts of the respective alkali metal or ammonium nitrates under anhydrous atmosphere, while lanthanum and cerium compounds have been crystallized from solutions in H_2_O or HNO_3_ (Guillou *et al.*, 1995 ▸, 1996 ▸; Gobichon *et al.*, 1999 ▸). For the heavier lanthanides, usually another structure type with a slightly different composition, namely in an *A*/*Ln* ratio of 2:1 instead of 3:2, is observed under similar reaction conditions (Manek & Meyer, 1992 ▸, 1993*a* ▸), and also for lithium, *e.g.* after the use of LiNO_3_ as a starting material, compounds with 2:1 ratio seem to be favoured (Manek & Meyer, 1993*b* ▸).

In this work a new member of this group of compounds, Ag_3_Dy_2_(NO_3_)_9_, is presented, the first one containing Ag and Dy, which has been found to crystallize in the above-mentioned structure type.

## 2. Structural commentary

Similar to many related compounds, the title compound was obtained from a melt of nitrates, in this case silver nitrate and dysprosium nitrate penta­hydrate. However, while for synthesis of related compounds, oxides are often used as lanthanide sources and the respective alkali metal nitrate or a eutectic combination of nitrates act as solvent as well as nitrate donor, in the present experimental setting the nitrates can be deployed in stoichiometric amounts. The crystals, which were found to be suitable for structure determination were obtained from a 2:1 mixture of Ag and Dy nitrates, *i.e.* a slight excess of AgNO_3_, as described in the experimental section. The surplus Ag is present as remaining AgNO_3_ as well as elemental silver after partial thermal or light-induced decomposition. So far, no hint of another compound with a 2:1 composition of metals in the Ag/Dy system, as could be expected for smaller lanthanides similar to the alkali metal or ammonium systems (Manek & Meyer, 1992 ▸, 1993*a* ▸), has been observed.

**Figure 1:**
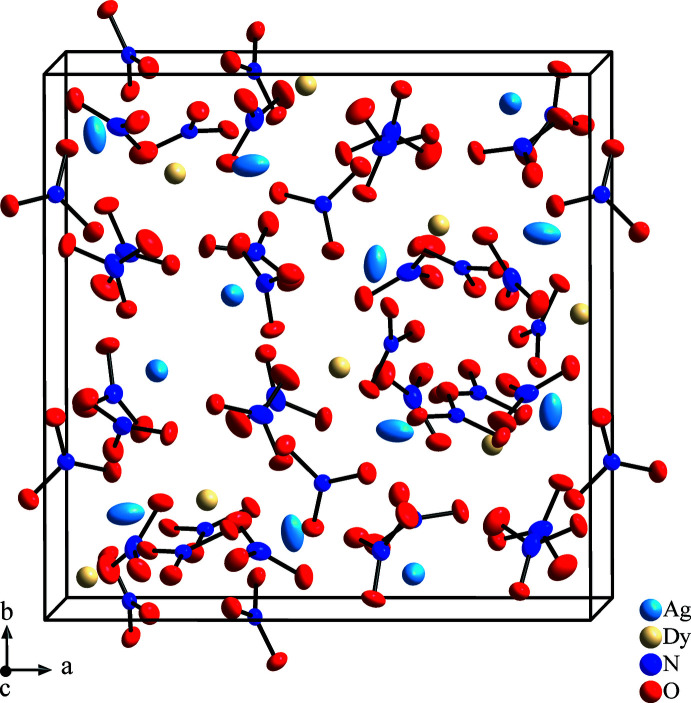
Unit cell of Ag_3_Dy_2_(NO_3_)_9_, view along the *c* axis, atomic displacement ellipsoids are drawn with a probability of 60%.

**Figure 2:**
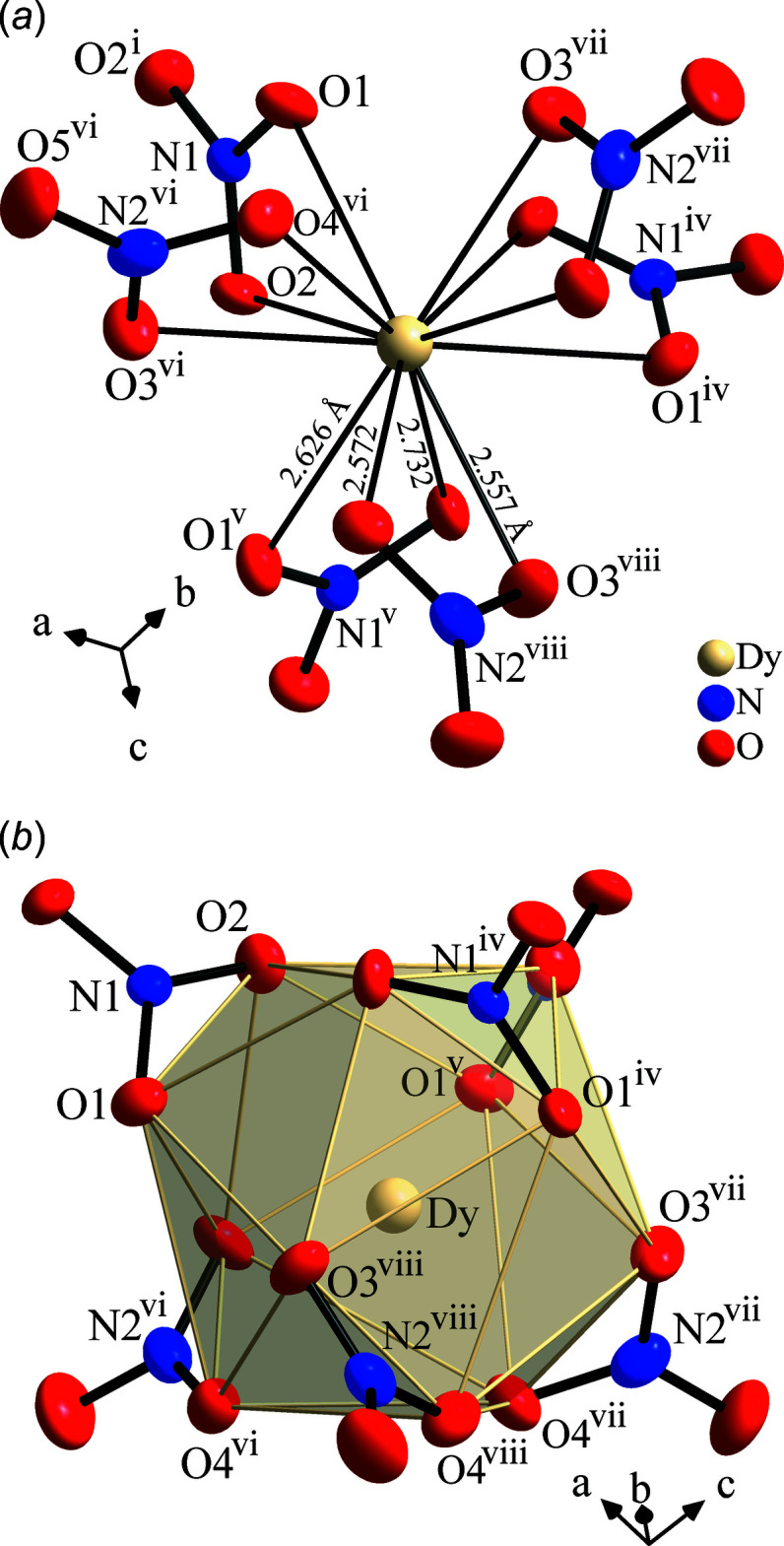
Twelvefold coordination of the Dy^3+^ ion by six bidentate nitrate ions in Ag_3_Dy_2_(NO_3_)_9_: (*a*) view along the threefold symmetry axis; (*b*) distorted icosa­hedron around Dy. Atoms are drawn at the 60% probability level. [Symmetry codes: (i) *z* + 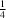 , −*y* + 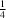 , *x* − 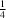 ; (iv) *x* − 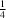 , *z* + 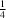 , −*y* + 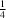 ; (v) −*y* + 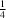 , *x* − 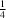 , *z* + 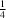 ; (vi) −*y* + 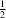 , −*z*, *x* − 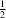 ; (vii) *x* − 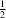 ; −*y* + 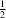 , −*z*; (viii) −*z*, *x* − 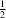 , −*y* + 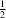 .]

**Figure 3:**
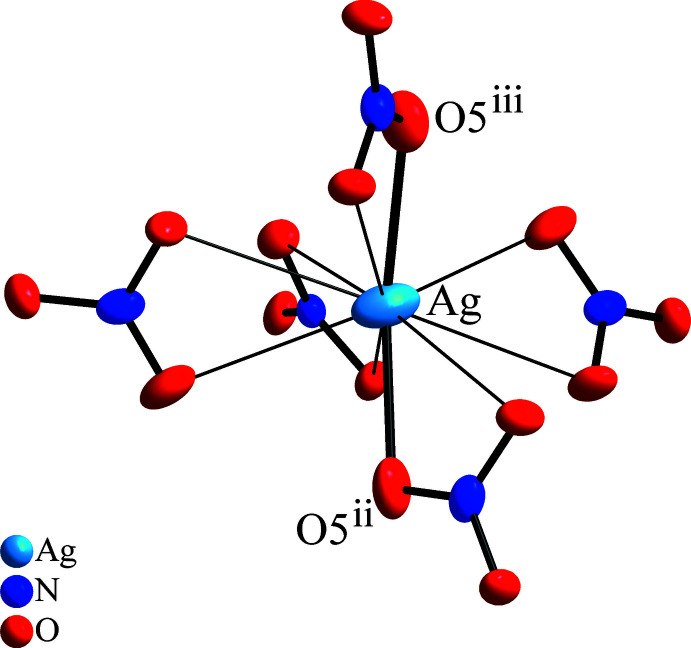
Coordination of the Ag^+^ cation by five bidentate nitrate anions. The shorter Ag—O bonds, which define the AgO_2_ dumbbell, are emphasized, displacement ellipsoids are drawn at the 60% probability level. [Symmetry codes: (ii) *y*, *z*, *x*; (iii) *x* + 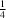 , −*z* + 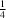 , *y* − 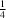 .]

**Figure 4:**
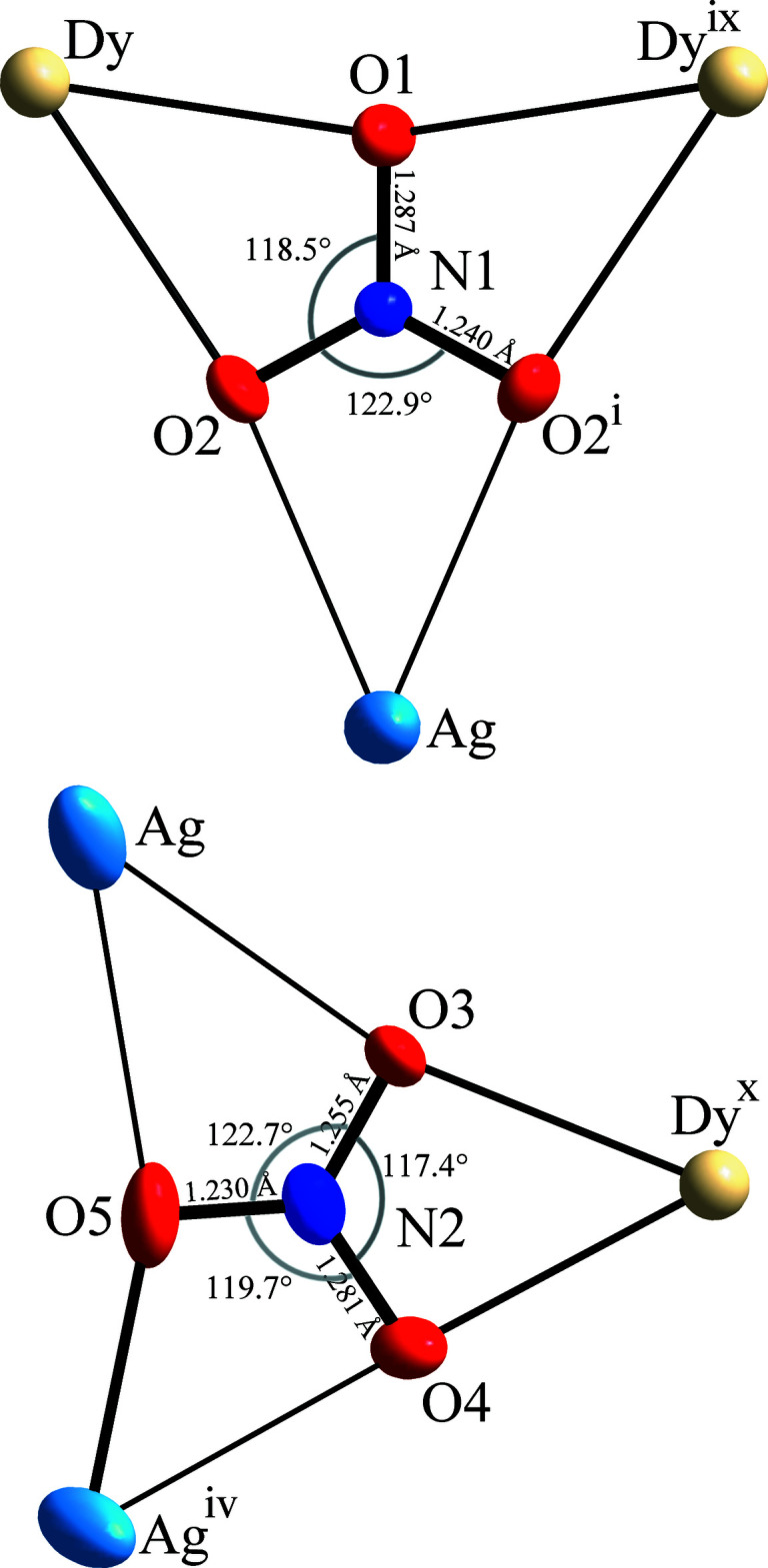
Planar surrounding of the two independent nitrate anions: NO_3_(1) (upper) coordinating two Dy and one Ag, view perpendicular to the twofold symmetry axis through Ag, N1, and O1; NO_3_(2) (lower) coordinating one Dy and two Ag, the short Ag—O5 bond is drawn thicker than other Ag—O bonds. All atoms are shown at the 60% probability level. [Symmetry codes: (i) *z* + 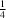 , −*y* + 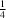 , *x* − 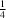 ; (iv) *x* − 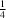 , *z* + 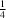 , −*y* + 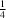 ; (ix) *y* + 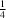 , −*x* + 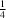 , *z* − 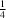 ; (*x*) *x* + 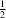 , −*y* + 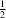 , −*z*.]

Ag_3_Dy_2_(NO_3_)_9_ (Fig. 1 ▸) crystallizes in space group *P*4_1_32 with most atoms at general positions except for Ag, N1 and O1 at 12*d* and Dy at 8*c* Wyckoff positions. The asymmetric unit comprises one Ag, one Dy, two N, and five O atoms. The Dy atom, being located on a threefold axis, is coordinated by six bidentate nitrate anions with Dy—O distances of 2.557 (11)–2.732 (11) Å (see Fig. 2 ▸ *a*), the surrounding oxygen atoms form a distorted icosa­hedron (Fig. 2 ▸ *b*). The polyhedra are connected to neighbouring icosa­hedra *via* common vertices, and inside this polyhedron the Dy atom is slightly off-centre, shown by formation of the shortest Dy—O distances to O3 and O4 as part of the same NO_3_^−^ anion (the lower one in Fig. 2 ▸ *b*), most probably driven by repulsion of next-neighbour Dy atoms. The silver atom is also coordinated by five nitrate ions in exclusively bidentate manner (Fig. 3 ▸). The Ag—O distances span quite a large range, so besides eight distances between 2.741 (11) and 3.004 (11) Å two relatively short distances of 2.383 (15) Å are found. These short bonds include oxygen atoms in almost opposite positions, which form an O—Ag—O angle of 154.7 (6)°, indicating the preferred formation of AgO_2_ dumbbells even in an environment of quite rigid complex anions, for instance observed in Ag_4_SiO_4_ (Klein & Jansen, 2008 ▸), in contrast to a more spherical ‘alkali metal-like’ coordination as in Ag_3_SbO_4_ (distorted rock salt structure; Klein & Jansen, 2010 ▸). Consequently, the Ag atom has its largest axis of the displacement ellipsoid perpendicular to the AgO_2_ dumbbell direction (see Fig. 3 ▸), which also represents the largest extension of an anisotropic parameter of all atoms in this structure (see supporting information, *U*_22_). The two independent nitrate ions are perfectly planar, with O—N—O angle sums of 360.00 and 359.79° around N1 and N2, respectively. Both the nitrate ions are situated between three bidentately coordinated metal atoms forming almost planar AgDy_2_(NO_3_) and Ag_2_Dy(NO_3_) units, respectively, as illustrated in Fig. 4 ▸. The longest N—O distances and the smallest O—N—O angles are found in the direction of coordinated Dy atoms, and in addition the Ag atom coordination, including a short Ag—O distance shows an O—N—O angle slightly below the mean value.

The appearance of this structure type for the combination Ag–Dy is somewhat remarkable. While silver as an atypical single-charged cation deforms its direct environment slightly to achieve a more convenient coordinative situation as explained above, dysprosium represents the heaviest lanthanide and, thus, the one with the smallest ionic radius observed in this structure type so far (Shannon, 1976 ▸), and a twelve-coordinate site seems to be unusual for this small lanthanide. This view is supported by the finding that compounds that include smaller lanthanide cations avoid to adopt this structure type in favour of another structure with a smaller coordination number and even a slightly different composition (*A*/*Ln* = 2:1; Manek & Meyer, 1992 ▸, 1993*a* ▸). Additionally, this might be confirmed by the ‘underbonding’ of the Dy cation, as the bond-valence sums (Brown & Altermatt, 1985 ▸) are calculated to be 2.51 valence units for the threefold positively charged ion, according to the parameters of Brese & O’Keeffe (1991 ▸).

The crystal structure has been qu­anti­tatively compared to isotypic structures by applying the program *compstru* (de la Flor *et al.*, 2016 ▸), available at the Bilbao Crystallographic Server (Aroyo *et al.*, 2006 ▸). With Ag_3_Dy_2_(NO_3_)_9_ as the reference structure, Table 1 ▸ lists the absolute distances between paired atoms as well as the arithmetic mean of the distance (*d*_av_) between paired atoms, the degree of lattice deviation (*S*) and the measure of similarity (*Δ*). Generally, the low values for *S* and *Δ* indicate a close relationship between all phases, including the trend to increasing numbers at larger differences of lattice parameters from Na to Rb compounds. The differences of *d*_av_, *S*, and *Δ* are of course determined in a higher degree by the more differing radii of the (more frequent) alkali metal cations than by those of the more similar lanthanide ions. Significantly, in all cases the largest displacements between atom pairs are observed for O5, *i.e.* the closest Ag-coordinating O atom, confirming the special bonding situation for Ag including the above-mentioned AgO_2_ dumbbells. Consequently, the whole NO_3_ anion, of which O5 is a part, is shifted slightly more than the atoms of the other anion. The Ag atom is also affected, as indicated by higher Ag—*A* displacements than those of the lanthanide cation pairs, while the coordination of the *Ln* cations remains similar (distortedly icosa­hedral, slightly off-centered), just accompanied by decreasing *Ln*—O distances with decreasing cation radii. An exception represents the, so far, only known Na structure, where the similarity as well as the relative displacements are about one order lower than for all other examples, indicating that the packing is distorted to a similar degree by the small Na cation as in the title compound by the Ag cation. However, the closest Ag—O distance is shorter than all Na—O distances in the related Na_3_Nd_2_(NO_3_)_9_.

## 3. Database survey

Several anhydrous rare-earth double nitrates of the composition *A*_3_ *Ln*_2_(NO_3_)_9_ have been investigated, mainly including larger lanthanide elements and alkali metals of medium size or ammonium cations, as listed in the *Chemical context*. Obviously, all of them seem to crystallize in the above-mentioned structure type, however, for some of them only the cubic lattice parameter is given. To date, detailed structural data are available for K_3_La_2_(NO_3_)_9_ (Gobichon *et al.*, 1999 ▸), K_3_Ce_2_(NO_3_)_9_ (Guillou *et al.*, 1995 ▸), Rb_3_Ce_2_(NO_3_)_9_ (Guillou *et al.*, 1996 ▸), K_3_Pr_2_(NO_3_)_9_ (Carnall *et al.*, 1973 ▸), Rb_3_Pr_2_(NO_3_)_9_ (Manek & Meyer, 1993*a* ▸), (NH_4_)_3_Pr_2_(NO_3_)_9_ (Manek & Meyer, 1992 ▸), Na_3_Nd_2_(NO_3_)_9_ (Stockhause & Meyer, 1997 ▸), K_3_Nd_2_(NO_3_)_9_ (Vigdorchik *et al.*, 1992 ▸), Rb_3_Nd_2_(NO_3_)_9_ (Vigdorchik *et al.*, 1990 ▸), and K_3_Bi_2_(NO_3_)_9_ (Goaz *et al.*, 2012 ▸).

## 4. Synthesis and crystallization

An alumina crucible was charged with 359 mg AgNO_3_ (2.1 mmol; Merck; p.A.) and 495 mg Dy(NO_3_)_3_·5H_2_O (1.1 mmol; Alfa Aesar; 99.99%). The mixture was melted together at 573 K for 72 h in an Ar atmosphere, and was cooled down to 453 K at a rate of 0.1 K min^−1^. Within an amorphous yellow–grey matrix, pale-yellow plates were found that were hygroscopic. EDX measurements on several crystals confirm the presence of Ag and Dy as the only elements heavier than oxygen. For the X-ray data collection, crystals were immersed into perfluoro­alkyl ether, which covers and acts as glue on a glass tip during the data collection at low temperatures.

## 5. Refinement

Crystal data, data collection and structure refinement details are summarized in Table 2 ▸. The structure was refined as an inversion twin.

## Supplementary Material

Crystal structure: contains datablock(s) global, I. DOI: 10.1107/S2056989023004747/pk2686sup1.cif

Structure factors: contains datablock(s) I. DOI: 10.1107/S2056989023004747/pk2686Isup2.hkl

CCDC reference: 2266445

Additional supporting information:  crystallographic information; 3D view; checkCIF report

## Figures and Tables

**Table 1:** Structure comparison of Ag_3_Dy_2_(NO_3_)_9_ with Na_3_Nd_2_(NO_3_)_9_^*a*^, K_3_Ce_2_(NO_3_)_9_^*b*^, K_3_Pr_2_(NO_3_)_9_^*c*,*g*^, Rb_3_Ce_2_(NO_3_)_9_^*d*^, Rb_3_Pr_2_(NO_3_)_9_^*e*,*g*^, and Rb_3_Nd_2_(NO_3_)_9_^*f*,*g*^, by using the program *Compstru* (de la Flor *et al.*, 2016 ▸) Cubic lattice parameters (Å), absolute atomic displacements (Å), arithmetic mean displacements (*d*_av_; Å), degree of lattice distortion (*S*), and measure of similarity (*Δ*)^*h*^.

*A* =	Na	K	K	Rb	Rb	Rb
*Ln* =	Nd	Ce	Pr	Ce	Pr	Nd
						
*a*	13.1279	13.5975	13.52	13.8411	13.8091	13.759
						
*A*	0.0035	0.3157	0.3325	0.4725	0.4680	0.4729
*Ln*	0.0151	0.2318	0.2440	0.3670	0.3768	0.3885
N1	0.0261	0.1605	0.1997	0.1848	0.2296	0.2352
O1	0.0187	0.2072	0.2035	0.2576	0.2912	0.3080
O2	0.0170	0.1786	0.1821	0.2763	0.2582	0.2646
N2	0.0223	0.3350	0.3555	0.5195	0.4883	0.4907
O3	0.0577	0.3059	0.3072	0.4321	0.4278	0.4502
O4	0.0346	0.3302	0.3271	0.4027	0.4815	0.4732
O5	0.0577	0.4583	0.4839	0.6160	0.6490	0.6483
						
*d_av_*	0.0320	0.2966	0.3080	0.4136	0.4280	0.4338
*S*	0.0032	0.0166	0.0135	0.0261	0.0249	0.0230
*Δ*	0.003	0.032	0.033	0.044	0.046	0.046

Notes: (*a*) Stockhause & Meyer (1997 ▸); (*b*) Guillou *et al.* (1995 ▸); (*c*) Carnall *et al.* (1973 ▸); (*d*) Guillou *et al.* (1996 ▸); (*e*) Manek & Meyer (1993*a* ▸); (*f*) Vigdorchik *et al.* (1990 ▸); (*g*) K_3_Pr_2_(NO_3_)_9_, Rb_3_Pr_2_(NO_3_)_9_, and Rb_3_Nd_2_(NO_3_)_9_ were originally described in *P*4_3_32 and were transformed into *P*4_1_32; (*h*) atom displacements are calculated by applying the lattice parameter of Ag_3_Dy_2_(NO_3_)_9_ [*a* = 13.2004 (4) Å] to the structure models of the listed compounds.

**Table 2:** Experimental details

Crystal data	
Chemical formula	Ag_3_Dy_2_(NO_3_)_9_
*M*_r_	1206.70
Crystal system, space group	Cubic, *P*4_1_32
Temperature (K)	223
*a* (Å)	13.2004 (4)
*V* (Å^3^)	2300.2 (2)
*Z*	4
Radiation type	Mo *K*α
μ (mm^−1^)	9.07
Crystal size (mm)	0.4 × 0.3 × 0.1

Data collection	
Diffractometer	Stoe StadiVari
Absorption correction	Empirical (using intensity measurements) (*X-AREA*; Stoe & Cie, 2015 ▸)
*T*_min_, *T*_max_	0.001, 0.215
No. of measured, independent and observed [*I* > 2σ(*I*)] reflections	37326, 763, 715
*R*_int_	0.159
(sin θ/λ)_max_ (Å^−1^)	0.617

Refinement	
*R*[*F* ^2^ > 2σ(*F* ^2^)], *wR*(*F* ^2^), *S*	0.045, 0.122, 1.18
No. of reflections	763
Δρ_max_, Δρ_min_ (e Å^−3^)	2.01, −1.07
Absolute structure	Refined as an inversion twin
Absolute structure parameter	0.18 (6)

Computer programs: *X-AREA* (Stoe & Cie, 2015 ▸), *SHELXS97*and *SHELX* (Sheldrick, 2008 ▸), *SHELXL2014/7* (Sheldrick, 2015 ▸), *DIAMOND* (Brandenburg & Putz (2018 ▸) and *publCIF* (Westrip, 2010 ▸).

## supplementary crystallographic information

### Crystal data

**Table:**

Ag_3_Dy_2_(NO_3_)_9_	Mo *K*α radiation, λ = 0.71073 Å
*M**_r_* = 1206.70	Cell parameters from 63387 reflections
Cubic, *P*4_1_32	θ = 2.2–30.7°
*a* = 13.2004 (4) Å	µ = 9.07 mm^−^^1^
*V* = 2300.2 (2) Å^3^	*T* = 223 K
*Z* = 4	Plate, yellow
*F*(000) = 2208	0.4 × 0.3 × 0.1 mm
*D*_x_ = 3.485 Mg m^−^^3^	

### Data collection

**Table:**

Stoe StadiVari diffractometer	763 independent reflections
Radiation source: Genix 3D HF Mo	715 reflections with *I* > 2σ(*I*)
Graded multilayer mirror monochromator	*R*_int_ = 0.159
Detector resolution: 5.81 pixels mm^-1^	θ_max_ = 26.0°, θ_min_ = 2.2°
ω scans	*h* = −16→16
Absorption correction: empirical (using intensity measurements) (X-AREA; Stoe & Cie, 2015)	*k* = −16→16
*T*_min_ = 0.001, *T*_max_ = 0.215	*l* = −16→16
37326 measured reflections	

### Refinement

**Table:**

Refinement on *F*^2^	Primary atom site location: structure-invariant direct methods
Least-squares matrix: full	Secondary atom site location: difference Fourier map
*R*[*F*^2^ > 2σ(*F*^2^)] = 0.045	*w* = 1/[σ^2^(*F*_o_^2^) + (0.0538*P*)^2^ + 51.0589*P*] where *P* = (*F*_o_^2^ + 2*F*_c_^2^)/3
*wR*(*F*^2^) = 0.122	(Δ/σ)_max_ < 0.001
*S* = 1.18	Δρ_max_ = 2.01 e Å^−^^3^
763 reflections	Δρ_min_ = −1.07 e Å^−^^3^
65 parameters	Absolute structure: Refined as an inversion twin
0 restraints	Absolute structure parameter: 0.18 (6)

### Special details

**Table:**

Geometry. All esds (except the esd in the dihedral angle between two l.s. planes) are estimated using the full covariance matrix. The cell esds are taken into account individually in the estimation of esds in distances, angles and torsion angles; correlations between esds in cell parameters are only used when they are defined by crystal symmetry. An approximate (isotropic) treatment of cell esds is used for estimating esds involving l.s. planes.
Refinement. Refined as a 2-component inversion twin.

### Fractional atomic coordinates and isotropic or equivalent isotropic displacement parameters (Å^2^)

**Table:**

	*x*	*y*	*z*	*U*_iso_*/*U*_eq_	
Ag	0.42059 (11)	0.1250	0.17059 (11)	0.0411 (6)	
Dy	0.03885 (6)	0.03885 (6)	0.03885 (6)	0.0233 (4)	
N1	0.2541 (9)	0.1250	0.0041 (9)	0.014 (4)	
O1	0.1852 (8)	0.1250	−0.0648 (8)	0.021 (3)	
O2	0.2346 (8)	0.0854 (8)	0.0870 (8)	0.022 (2)	
N2	0.3881 (11)	0.3676 (11)	0.1011 (10)	0.023 (3)	
O3	0.4747 (8)	0.3304 (9)	0.0888 (9)	0.025 (2)	
O4	0.3674 (8)	0.4493 (8)	0.0530 (9)	0.024 (2)	
O5	0.3217 (10)	0.3253 (11)	0.1508 (9)	0.035 (3)	

### Atomic displacement parameters (Å^2^)

**Table:**

	*U*^11^	*U*^22^	*U*^33^	*U*^12^	*U*^13^	*U*^23^
Ag	0.0260 (7)	0.0713 (17)	0.0260 (7)	−0.0052 (7)	−0.0006 (8)	0.0052 (7)
Dy	0.0233 (4)	0.0233 (4)	0.0233 (4)	0.0002 (3)	0.0002 (3)	0.0002 (3)
N1	0.015 (5)	0.011 (8)	0.015 (5)	0.000 (4)	−0.001 (7)	0.000 (4)
O1	0.022 (5)	0.017 (7)	0.022 (5)	−0.005 (4)	−0.003 (6)	0.005 (4)
O2	0.026 (6)	0.022 (5)	0.018 (5)	0.001 (4)	0.000 (5)	0.007 (4)
N2	0.019 (7)	0.031 (8)	0.018 (7)	−0.006 (6)	0.001 (5)	−0.004 (6)
O3	0.015 (5)	0.030 (6)	0.029 (6)	0.005 (5)	0.005 (5)	0.008 (5)
O4	0.022 (6)	0.017 (5)	0.033 (7)	0.001 (5)	−0.001 (5)	0.000 (5)
O5	0.036 (7)	0.043 (8)	0.027 (6)	−0.014 (6)	0.008 (6)	0.007 (6)

### Geometric parameters (Å, º)

**Table:**

Ag—O5^i^	2.383 (15)	Dy—O1^i^	2.626 (2)
Ag—O5^ii^	2.383 (15)	Dy—O1^vii^	2.626 (2)
Ag—O2^iii^	2.741 (11)	Dy—O1	2.626 (2)
Ag—O2	2.741 (11)	Dy—O2	2.732 (11)
Ag—O4^ii^	2.793 (11)	Dy—O2^vii^	2.732 (11)
Ag—O4^i^	2.793 (11)	Dy—O2^i^	2.732 (11)
Ag—O5^iii^	2.960 (15)	N1—O2	1.240 (14)
Ag—O5	2.960 (15)	N1—O2^iii^	1.240 (14)
Ag—N2^ii^	2.972 (14)	N1—O1	1.29 (2)
Ag—N2^i^	2.972 (14)	O1—Dy^viii^	2.626 (2)
Ag—O3	3.004 (11)	N2—O5	1.230 (17)
Ag—O3^iii^	3.004 (11)	N2—O3	1.255 (18)
Dy—O3^iv^	2.557 (11)	N2—O4	1.281 (18)
Dy—O3^v^	2.557 (11)	N2—Ag^vii^	2.972 (14)
Dy—O3^vi^	2.557 (11)	O3—Dy^ix^	2.557 (11)
Dy—O4^vi^	2.572 (11)	O4—Dy^ix^	2.572 (11)
Dy—O4^iv^	2.572 (11)	O4—Ag^vii^	2.793 (11)
Dy—O4^v^	2.572 (11)	O5—Ag^vii^	2.383 (15)
			
O5^i^—Ag—O5^ii^	154.7 (6)	O3^vi^—Dy—O4^v^	118.6 (3)
O5^i^—Ag—O2^iii^	120.7 (4)	O4^vi^—Dy—O4^v^	70.6 (4)
O5^ii^—Ag—O2^iii^	83.8 (4)	O4^iv^—Dy—O4^v^	70.6 (4)
O5^i^—Ag—O2	83.8 (4)	O3^iv^—Dy—O1^i^	66.9 (4)
O5^ii^—Ag—O2	120.7 (4)	O3^v^—Dy—O1^i^	67.4 (3)
O2^iii^—Ag—O2	46.8 (4)	O3^vi^—Dy—O1^i^	168.5 (4)
O5^i^—Ag—O4^ii^	109.2 (4)	O4^vi^—Dy—O1^i^	139.4 (2)
O5^ii^—Ag—O4^ii^	48.8 (3)	O4^iv^—Dy—O1^i^	112.0 (4)
O2^iii^—Ag—O4^ii^	115.5 (3)	O4^v^—Dy—O1^i^	72.6 (3)
O2—Ag—O4^ii^	162.3 (3)	O3^iv^—Dy—O1^vii^	168.5 (4)
O5^i^—Ag—O4^i^	48.8 (3)	O3^v^—Dy—O1^vii^	66.9 (4)
O5^ii^—Ag—O4^i^	109.2 (4)	O3^vi^—Dy—O1^vii^	67.4 (3)
O2^iii^—Ag—O4^i^	162.3 (3)	O4^vi^—Dy—O1^vii^	72.6 (3)
O2—Ag—O4^i^	115.5 (3)	O4^iv^—Dy—O1^vii^	139.4 (2)
O4^ii^—Ag—O4^i^	82.1 (5)	O4^v^—Dy—O1^vii^	112.0 (4)
O5^i^—Ag—O5^iii^	116.8 (5)	O1^i^—Dy—O1^vii^	106.9 (3)
O5^ii^—Ag—O5^iii^	73.3 (5)	O3^iv^—Dy—O1	67.4 (3)
O2^iii^—Ag—O5^iii^	74.9 (3)	O3^v^—Dy—O1	168.5 (4)
O2—Ag—O5^iii^	64.8 (3)	O3^vi^—Dy—O1	66.9 (4)
O4^ii^—Ag—O5^iii^	116.5 (3)	O4^vi^—Dy—O1	112.0 (4)
O4^i^—Ag—O5^iii^	96.8 (3)	O4^iv^—Dy—O1	72.6 (3)
O5^i^—Ag—O5	73.3 (5)	O4^v^—Dy—O1	139.4 (2)
O5^ii^—Ag—O5	116.8 (5)	O1^i^—Dy—O1	106.9 (3)
O2^iii^—Ag—O5	64.8 (3)	O1^vii^—Dy—O1	106.9 (3)
O2—Ag—O5	74.9 (3)	O3^iv^—Dy—O2	65.6 (4)
O4^ii^—Ag—O5	96.8 (3)	O3^v^—Dy—O2	122.7 (4)
O4^i^—Ag—O5	116.5 (3)	O3^vi^—Dy—O2	108.3 (3)
O5^iii^—Ag—O5	136.1 (5)	O4^vi^—Dy—O2	158.2 (3)
O5^i^—Ag—N2^ii^	133.8 (4)	O4^iv^—Dy—O2	104.7 (3)
O5^ii^—Ag—N2^ii^	23.4 (4)	O4^v^—Dy—O2	129.0 (4)
O2^iii^—Ag—N2^ii^	99.1 (3)	O1^i^—Dy—O2	62.4 (2)
O2—Ag—N2^ii^	142.2 (4)	O1^vii^—Dy—O2	103.1 (4)
O4^ii^—Ag—N2^ii^	25.4 (3)	O1—Dy—O2	47.8 (4)
O4^i^—Ag—N2^ii^	97.1 (3)	O3^iv^—Dy—O2^vii^	122.7 (4)
O5^iii^—Ag—N2^ii^	94.4 (4)	O3^v^—Dy—O2^vii^	108.3 (3)
O5—Ag—N2^ii^	108.1 (3)	O3^vi^—Dy—O2^vii^	65.6 (4)
O5^i^—Ag—N2^i^	23.4 (4)	O4^vi^—Dy—O2^vii^	104.7 (3)
O5^ii^—Ag—N2^i^	133.8 (4)	O4^iv^—Dy—O2^vii^	129.0 (4)
O2^iii^—Ag—N2^i^	142.2 (4)	O4^v^—Dy—O2^vii^	158.2 (3)
O2—Ag—N2^i^	99.1 (3)	O1^i^—Dy—O2^vii^	103.1 (4)
O4^ii^—Ag—N2^i^	97.1 (3)	O1^vii^—Dy—O2^vii^	47.8 (4)
O4^i^—Ag—N2^i^	25.4 (3)	O1—Dy—O2^vii^	62.4 (2)
O5^iii^—Ag—N2^i^	108.1 (3)	O2—Dy—O2^vii^	60.9 (4)
O5—Ag—N2^i^	94.4 (4)	O3^iv^—Dy—O2^i^	108.3 (3)
N2^ii^—Ag—N2^i^	117.8 (5)	O3^v^—Dy—O2^i^	65.6 (4)
O5^i^—Ag—O3	107.3 (4)	O3^vi^—Dy—O2^i^	122.7 (4)
O5^ii^—Ag—O3	75.0 (4)	O4^vi^—Dy—O2^i^	129.0 (4)
O2^iii^—Ag—O3	66.5 (3)	O4^iv^—Dy—O2^i^	158.2 (3)
O2—Ag—O3	103.9 (3)	O4^v^—Dy—O2^i^	104.7 (3)
O4^ii^—Ag—O3	61.3 (3)	O1^i^—Dy—O2^i^	47.8 (4)
O4^i^—Ag—O3	127.5 (3)	O1^vii^—Dy—O2^i^	62.4 (2)
O5^iii^—Ag—O3	132.0 (3)	O1—Dy—O2^i^	103.1 (4)
O5—Ag—O3	42.9 (3)	O2—Dy—O2^i^	60.9 (4)
N2^ii^—Ag—O3	65.8 (3)	O2^vii^—Dy—O2^i^	60.9 (4)
N2^i^—Ag—O3	119.9 (4)	O2—N1—O2^iii^	122.9 (18)
O5^i^—Ag—O3^iii^	75.0 (4)	O2—N1—O1	118.5 (9)
O5^ii^—Ag—O3^iii^	107.3 (4)	O2^iii^—N1—O1	118.5 (9)
O2^iii^—Ag—O3^iii^	103.9 (3)	N1—O1—Dy	98.7 (3)
O2—Ag—O3^iii^	66.5 (3)	N1—O1—Dy^viii^	98.7 (3)
O4^ii^—Ag—O3^iii^	127.5 (3)	Dy—O1—Dy^viii^	162.6 (6)
O4^i^—Ag—O3^iii^	61.3 (3)	N1—O2—Dy	94.9 (9)
O5^iii^—Ag—O3^iii^	42.9 (3)	N1—O2—Ag	95.1 (9)
O5—Ag—O3^iii^	132.0 (3)	Dy—O2—Ag	169.6 (5)
N2^ii^—Ag—O3^iii^	119.9 (4)	O5—N2—O3	122.7 (15)
N2^i^—Ag—O3^iii^	65.8 (3)	O5—N2—O4	119.7 (14)
O3—Ag—O3^iii^	170.1 (5)	O3—N2—O4	117.4 (13)
O3^iv^—Dy—O3^v^	116.77 (16)	O5—N2—Ag^vii^	50.3 (9)
O3^iv^—Dy—O3^vi^	116.77 (16)	O3—N2—Ag^vii^	170.4 (11)
O3^v^—Dy—O3^vi^	116.77 (16)	O4—N2—Ag^vii^	69.4 (8)
O3^iv^—Dy—O4^vi^	118.6 (3)	N2—O3—Dy^ix^	97.0 (9)
O3^v^—Dy—O4^vi^	76.0 (4)	N2—O3—Ag	95.2 (9)
O3^vi^—Dy—O4^vi^	50.0 (3)	Dy^ix^—O3—Ag	157.7 (5)
O3^iv^—Dy—O4^iv^	50.0 (3)	N2—O4—Dy^ix^	95.6 (8)
O3^v^—Dy—O4^iv^	118.6 (3)	N2—O4—Ag^vii^	85.1 (8)
O3^vi^—Dy—O4^iv^	76.0 (4)	Dy^ix^—O4—Ag^vii^	170.9 (5)
O4^vi^—Dy—O4^iv^	70.6 (4)	N2—O5—Ag^vii^	106.3 (11)
O3^iv^—Dy—O4^v^	76.0 (4)	N2—O5—Ag	98.0 (11)
O3^v^—Dy—O4^v^	50.0 (3)	Ag^vii^—O5—Ag	148.6 (5)

Symmetry codes: (i) *y*, *z*, *x*; (ii) *x*+1/4, −*z*+1/4, *y*−1/4; (iii) *z*+1/4, −*y*+1/4, *x*−1/4; (iv) −*y*+1/2, −*z*, *x*−1/2; (v) −*z*, *x*−1/2, −*y*+1/2; (vi) *x*−1/2, −*y*+1/2, −*z*; (vii) *z*, *x*, *y*; (viii) *y*+1/4, −*x*+1/4, *z*−1/4; (ix) *x*+1/2, −*y*+1/2, −*z*.
